# Supplementary material for: Eco-Efficient Quantification of Glucosinolates in Camelina Seed, Oil, and Defatted Meal: Optimization, Development, and Validation of a UPLC-DAD Method
Source: Antioxidants (Basel). 2022 Dec 10;11(12):2441. doi: 10.3390/antiox11122441 (PMC9774283; doi:10.3390/antiox11122441)
Supplement: Supplementary file 1 [file antioxidants-11-02441-s001.zip › antioxidants-2070761-supplementary.pdf]

Supplementary document for manuscript Antioxidants-2070761

Abbreviations used in the Supplementary document:

Std.: Standard

Diff.: Difference

Blk.: Blank

Inf.: Interference

N/A: Not available

Est.: Estimated

LOQ: Limit of Quantitation

LOD: Limit of Detection

Avg.: Average

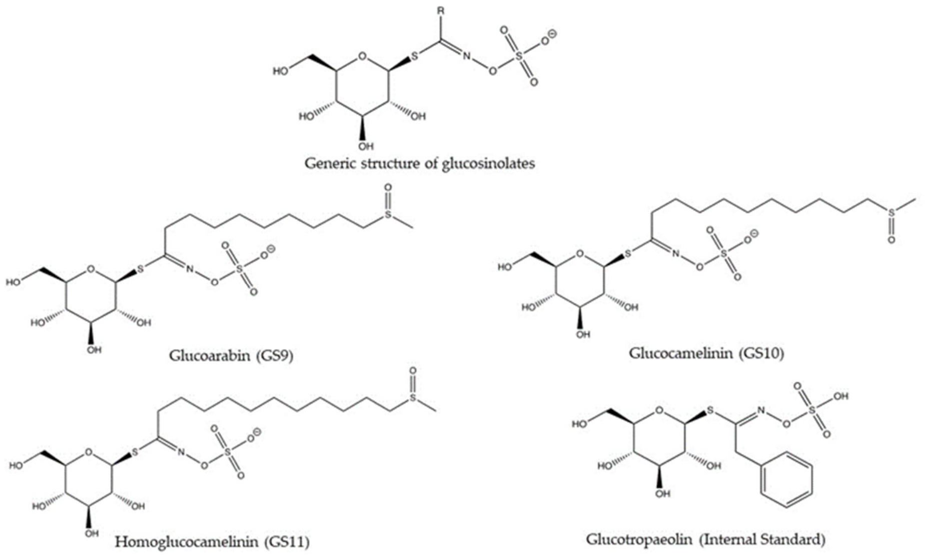

Figure S1. Chemical structures for general glucosinolates, camelina glucosinolates, and glucotropaeolin (internal standard).

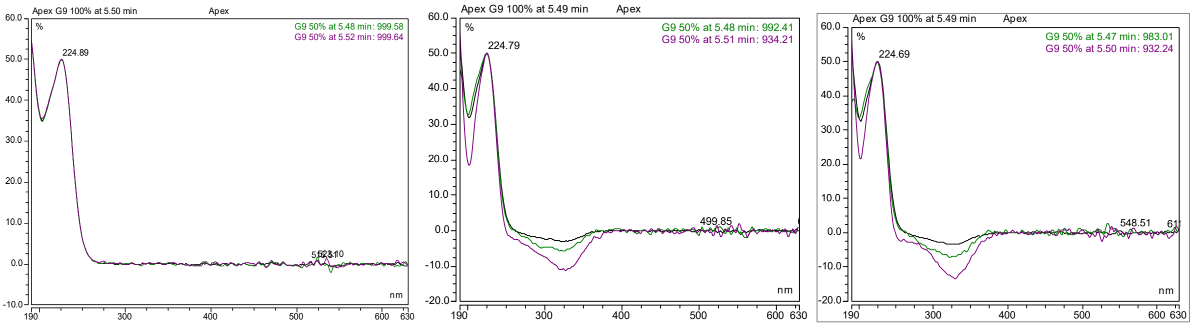

Figure S2. UV-vis spectrum of Compound Glucoarabin (GS9) in reference standard solution (left), Validation study sample #1 (camelina seed) (mid) and spiked Placebo sample #1 (canola seed) (right).

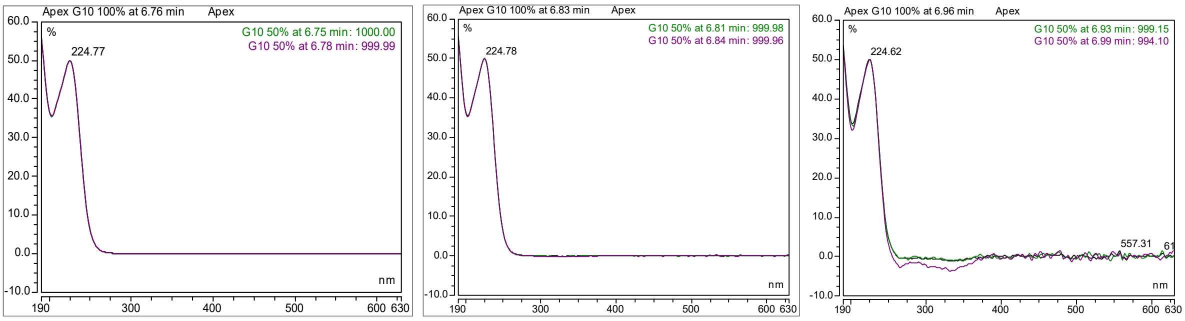

Figure S3. UV-vis spectrum of Compound Glucocamelinin (GS10) in reference standard solution (left), Validation study sample #1 (camelina seed) (mid) and spiked Placebo sample #1 (canola seed) (right).

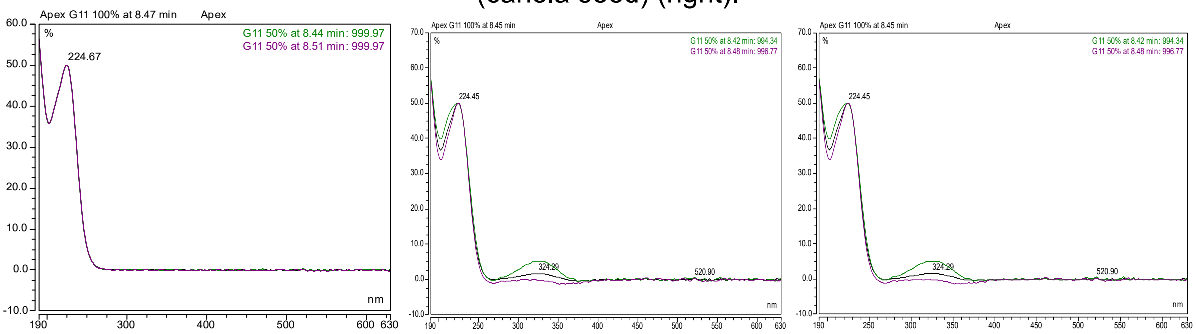

Figure S4. UV-vis spectrum of Compound 11-Methylsulfinylundecylglucosinolate (GS11) in reference standard solution (left), Validation study sample #1 (camelina seed) (mid) and spiked Placebo sample #1 (canola seed) (right).

Table S1: Specificity – Camelina Seed

Compound	Retention Time (min)					Peak Area				
	Spiked Placebo	Sample	Std	%Diff. - Placebo	%Diff. - Sample	Blk	Placebo	Smallest Std	%Inf. - blk	%Inf. - Placebo
Internal Std	3.12	3.12	3.12	0.128	0.160	0.000	0.000	0.010	0.0	0.0
GS9	5.58	5.59	5.51	1.335	1.496	0.000	0.000	0.016	0.0	0.0
GS10	6.83	6.84	6.86	0.541	0.380	0.000	0.000	0.010	0.0	0.0
GS11	8.41	8.43	8.44	0.427	0.178	0.000	0.000	0.015	0.0	0.0

Table S2: Specificity – Camelina Oil

Compound	Retention Time (min)					Peak Area				
	Spiked Placebo	Sample	Std	%Diff. - Placebo	%Diff. - Sample	Blk	Placebo	Smallest Std	%Inf. - blk	%Inf. - Placebo
Internal Std	3.13	N/A	3.15	0.606	N/A	0.000	0.000	0.010	0.0	0.0
GS9	5.51	N/A	5.51	0.018	N/A	0.000	0.000	0.016	0.0	0.0
GS10	6.88	N/A	6.86	0.262	N/A	0.000	0.000	0.010	0.0	0.0
GS11	8.50	N/A	8.44	0.614	N/A	0.000	0.000	0.015	0.0	0.0

Table S3: Specificity – Camelina Defatted Meal

Compound	Retention Time (min)					Peak Area				
	Spiked Placebo	Sample	Std	%Diff. - Placebo	%Diff. - Sample	Blk	Placebo	Smallest Std	%Inf. - blk	%Inf. - Placebo
Internal Std	3.13	3.12	3.12	0.480	0.000	0.000	0.000	0.010	0.0	0.0
GS9	5.48	5.49	5.51	0.419	0.218	0.000	0.000	0.016	0.0	0.0
GS10	6.82	6.82	6.86	0.687	0.570	0.000	0.000	0.010	0.0	0.0
GS11	8.40	8.42	8.44	0.570	0.344	0.000	0.000	0.015	0.0	0.0

Table S4: System Suitability

Glucotropaeolin (Internal Std)								
Injection	Peak Area	Peak Area %RSD	Retention Time (min)	Retention Time %RSD	Tailing Factor	Average	Plate Count	Average
1	0.270		3.022		1.29		11,139	
2	0.271		3.017		1.32		11,269	
3	0.272	0.62	3.016	0.15	1.33	1.32	11,569	11,347.60
4	0.269		3.011		1.33		11,434	
5	0.268		3.011		1.32		11,327	
Glucoarabin (GS9)								
Injection	Peak Area	Peak Area %RSD	Retention Time (min)	Retention Time %RSD	Tailing Factor	Average	Plate Count	Average
1	0.195		5.434		1.34		110,543	
2	0.197		5.432		1.34		115,985	
3	0.199	1.08	5.438	0.06	1.39	1.36	113,271	115,122.20
4	0.195		5.433		1.35		117,229	
5	0.194		5.429		1.38		118,583	
Glucocamelinin (GS10)								

Injection	Peak Area	Peak Area %RSD	Retention Time (min)	Retention Time %RSD	Tailing Factor	Average	Plate Count	Average
1	0.816	0.73	6.789	0.07	1.43	1.46	159,291	162,732.40
2	0.828		6.789		1.46		159,573	
3	0.825		6.789		1.49		163,265	
4	0.815		6.782		1.47		163,039	
5	0.817		6.780		1.47		168,494	
11-Methylsulfinylundecylglucosinolate (GS11)								
Injection	Peak Area	Peak Area %RSD	Retention Time (min)	Retention Time %RSD	Tailing Factor	Average	Plate Count	Average
1	0.218	1.29	8.349	0.05	1.33	1.39	149,373	154,168.60
2	0.224		8.347		1.40		152,901	
3	0.220		8.340		1.41		155,081	
4	0.224		8.345		1.39		159,938	
5	0.223		8.341		1.40		153,550	

Table S5: **Linearity**

Compound	Concentration (mcg/mL)					R Square	Regression equation
	Std1	Std2	Std3	Std4	Std5		
Glucotropaeolin (IS)	2.5573	2.5573	2.5573	2.5573	2.5573	N/A	N/A
Glucoarabin (GS9)	0.6664	1.3328	3.3320	6.6640	29.9880	0.99997	y = 24.51645x-1.34188
Glucocamelinin (GS10)	1.0702	2.1403	5.3508	10.7016	48.1572	0.99997	y = 35.89640x-2.40103
11-Methylsulfinylundecylglucosinolate (GS11)	0.5112	1.0224	2.5560	5.1120	23.0040	0.99997	y = 24.41998x-0.67717

Table S6: LOQ, LOD and Range

Compound	Concentration (mcg/mL)	S/N Ratio	Est. LOQ (mcg/mL)	Est. LOD (mcg/mL)	Range (mcg/g)
GS9	0.300	14.6	0.300	0.090	(141.84, 2243.01)
GS10	0.188	10.5	0.188	0.056	(229.28, 11371.85)
GS11	0.266	17.7	0.266	0.080	(106.14, 7313.98)

Table S7: Robustness

Concentration (mcg/mL)

Robustness # 1 Mobile Phase

Component	Condition	Injection 1	Injection 2	Injection 3	Injection 4	Injection 5	Avg.	% Difference
GS9	Original (0.1% PA)	2.560	2.551	2.559	2.569	2.539	2.556	
	0.095% PA	2.573	2.605	2.570	2.620	2.442	2.562	0.25
	0.105% PA	2.650	2.623	2.606	2.446	2.496	2.564	0.34
GS10	Original (0.1% PA)	30.186	29.949	29.902	29.779	30.138	29.991	
	0.095% PA	29.566	29.049	29.929	29.048	31.499	29.818	0.57

	0.105% PA	28.898	29.308	29.291	31.315	31.494	30.061	0.24
GS11	Original (0.1% PA)	23.091	22.996	22.982	22.861	23.152	23.017	
	0.095% PA	22.853	22.444	22.784	22.472	23.925	22.896	0.53
	0.105% PA	22.090	22.480	22.454	24.101	24.292	23.083	0.29
Robustness # 2 Column Temperature								
Component	Condition	Injection 1	Injection 2	Injection 3	Injection 4	Injection 5	Avg.	% Difference
GS9	Original (45°C)	3.200	3.218	3.249	3.208	3.210	3.217	
	43°C	3.240	3.192	3.247	3.234	3.208	3.224	0.24
	48°C	3.212	3.184	3.197	3.235	3.178	3.201	0.48
GS10	Original (45°C)	15.410	15.580	15.494	15.471	15.563	15.504	
	43°C	15.488	15.387	15.635	15.636	15.490	15.527	0.15
	48°C	15.633	15.374	15.452	15.688	15.439	15.517	0.09
GS11	Original (45°C)	4.223	4.336	4.237	4.359	4.367	4.304	
	43°C	4.312	4.256	4.313	4.364	4.296	4.308	0.09
	48°C	4.270	4.262	4.385	4.368	4.295	4.316	0.28
Robustness # 3 Flow Rate								
Component	Condition	Injection 1	Injection 2	Injection 3	Injection 4	Injection 5	Avg.	% Difference
GS9	Original (0.4 ml/min)	3.199	3.200	3.218	3.249	3.208	3.215	
	0.38 ml/min	3.202	3.239	3.171	3.219	3.221	3.210	0.13
	0.42 ml/min	3.206	3.249	3.256	3.215	3.219	3.229	0.44
GS10	Original (0.4 ml/min)	15.422	15.410	15.580	15.494	15.471	15.476	
	0.38 ml/min	15.275	15.419	15.337	15.517	15.561	15.422	0.35
	0.42 ml/min	15.380	15.538	15.546	15.458	15.896	15.563	0.57
GS11	Original (0.4 ml/min)	4.219	4.223	4.336	4.237	4.359	4.275	
	0.38 ml/min	4.255	4.312	4.278	4.276	4.337	4.292	0.40
	0.42 ml/min	4.323	4.275	4.255	4.292	4.271	4.283	0.20
